# Prevalence of and factors associated with a treatment delay due to the COVID-19 pandemic in patients with gastrointestinal cancer in Europe

**DOI:** 10.1007/s00432-023-05062-w

**Published:** 2023-07-06

**Authors:** Christoph Roderburg, Sven H. Loosen, Catherine Leyh, Markus S. Joerdens, Raphael Mohr, Tom Luedde, Svetlana Alymova, Isabel Klein, Karel Kostev

**Affiliations:** 1 Department of Gastroenterology, Hepatology and Infectious Diseases, University Hospital Düsseldorf, Medical Faculty of Heinrich Heine University Düsseldorf, Moorenstrasse 5, 40225 Düsseldorf, Germany; 2 Department of Hepatology and Gastroenterology, Charité University Medicine Berlin, Augustenburger Platz 1, 13353 Berlin, Germany; 3 Oncology, Real World Solutions, IQVIA, Frankfurt, Germany; 4 Epidemiology, IQVIA, Frankfurt, Germany

**Keywords:** SARS-CoV-2, Tumor, Colorectal cancer, Malignancy, Pandemic

## Abstract

**Background:**

Recent studies have raised the issue of delayed cancer care during the COVID-19 pandemic, but the extent of delays and cancellations in cancer treatment, screening and diagnosis varied widely by geographic region and study design, highlighting the need for further research.

**Methods:**

We used the Oncology Dynamics (OD) database featuring data from a cross-sectional, partially retrospective survey to analyze treatment delays in 30,171 GI cancer patients from five European countries (Germany, France, UK, Spain, and Italy). Risk factors for treatment delays were identified using multivariable logistic regression models.

**Results:**

Treatment delays were documented in 1342 (4.5%) of the study patients, with most patients having a delay of less than 3 months (3.2%). We observed decisive differences of treatment delay in relation to geographical, healthcare- and patient-related factors. Treatment delay was highest in France (6.7%) and Italy (6.5%) and lowest in Spain (1.9%, p < 0.001). 5.9% of patients treated at general hospitals but only 1.9% of those treated by office-based physicians experienced treatment delays (p < 0.001). Moreover, the difference between lines of therapy was highly significant and ranged from 7.2% for early-stage patients in primary therapy to 2.6% in advanced/metastatic cancer patients receiving 4th or later line therapy (p < 0.001). Finally, the proportion of cases with delayed treatments increased from 3.5% in asymptomatic patients (ECOG 0) to 9.9% in bedridden patients (ECOG IV, p < 0.001). Results were confirmed in multivariable logistic regression models.

**Summary:**

Our data highlight the problem of delayed treatment of tumor patients in the context of the COVID-19 pandemic. Identified risk factors for delayed treatment, such as poor general health or treatment in smaller hospitals, offer starting points for future concepts of “pandemic preparedness”.

## Introduction

Globally, as of April 19, 2023, there have been 763,740,140 confirmed cases of COVID-19, including 6,908,554 deaths, reported to the WHO (2023). COVID-19 is caused by the severe acute respiratory syndrome coronavirus-2 (SARS-CoV-2) and involves pulmonary (e.g., rhinorrhea, cough, and dyspnea) as well as extra-pulmonary symptoms (e.g., anosmia, dizziness, and nausea/vomiting) (Meyer et al. 2021). Intensive vaccination campaigns, non-pharmacological interventions and the development of numerous drugs have recently reduced infection rates and mortality (Meyer et al. 2021).

The COVID-19 pandemic has posed major challenges to health care systems and human societies worldwide (Assefa et al. 2021). During the pandemic, multiple measures were taken in Europe and in large parts of the rest of the world to reduce the number of infections and mortality (Gianicolo et al. 2020; Grote et al. 2021). In Europe, these included legally defined contact and travel restrictions, the compulsory use of masks for large parts of the public life and specific hygiene measures (Gianicolo et al. 2020). Similarly, the COVID-19 pandemic has had a profound impact on healthcare facilities and the way in which patients access care. The implementation of restriction measures, such as visitor restrictions, personal protective equipment requirements, and the restructuring of services, has significantly limited the consultation process for patients in healthcare facilities. This means that patients with non-urgent medical needs may have to face longer wait times or difficulties accessing care altogether.

As the peak of the pandemic has passed, the long-term sequela of the SARS-CoV-2 infection, such as delayed cancer diagnoses and/or clinical problems derived from delayed cancer treatments dominate the medical and scientific dialogue. However, the quality and scale of the COVID-19 restriction measures were highly different between different European countries, suggesting that the impact of COVID-19 restriction measures, i.e. the consequences of delayed cancer treatments, might have affected patients in different European countries to varying extents (Iftekhar et al. 2021).

In the present manuscript, we used data from IQVIA’s Oncology Dynamics (OD) database (Alymova et al. 2022), one of the largest survey panel data in Europe, to study the prevalence and associated factors of treatment delays due to COVID-19 pandemic among patients with gastrointestinal cancers in Europe.

## Methods

### Database

This retrospective cross-sectional study is based on the data from IQVIA’s Oncology Dynamics (OD) database (Alymova et al. 2022). This source is a cross-sectional, partially retrospective survey collecting anonymized patient cases from a representative panel of oncologists. OD collects fully anonymized patient-level data on drug-treated cancer cases in several countries worldwide. Data collection and reporting is conducted through a standardized online questionnaire where all items are mandatory. A reporting manual with precise instructions on completing the questionnaire is provided to each respondent. Specific instructions are displayed through a ‘pop-up’ system throughout the survey to provide clear definitions for the desired variables. Physicians are also asked to enter factual information from the patient medical record to avoid recall bias. Further tactics to ensure input accuracy include controlled code lists and multiple-choice questions as well as interactive filters that limit non-applicable questions (e.g., items on cancer-specific biomarkers). Responses are immediately validated against previous answers and reference files; “unexpected value” messages are displayed to the participant, prompting them to double-check their response. Physicians are instructed to report the most recent consecutive cases (up to 30 cases depending on the specialty) they had treated during the last 7-day period to discourage selective case submission. After the form submission, additional validations and trend checks are performed; anomalous values are discussed with the submitting participant and corrected as needed.

### Patient selection, study outcome, and variables

Surveys of all patients with gastrointestinal cancers (GIST, colorectal cancer, gastric cancer, pancreatic cancer, liver cancer and esophageal cancer) completed between January 1, 2021 and December 31, 2022 were available for five European countries: Germany, France, Italy, the United Kingdom (UK), and Spain. The main outcome of the study was the prevalence of treatment delays due to COVID-19 pandemic depending on different factors including country, cancer site, age group, sex, treating facility (academic cancer facility, non-academic cancer facility, general hospital, office based practitioner), current stage, site of metastasis (liver, peritoneum, lung) and ECOG performance status (asymptomatic, symptomatic fully ambulatory, symptomatic in bed less than 50%, symptomatic in bed greater than 50% and bedridden). Information on therapy delay was available in five categories: postponed by less than 3, 3–6 or > 6 months.

### Statistical analysis

Baseline and clinical characteristics were compared between patients with and without therapy delay using a χ^2^ test. Multivariable logistic regression models were conducted to investigate the association between demographic and clinical variables and therapy delay (yes versus no). p values < 0.05 were considered statistically significant. All analyses were performed using SAS 9.4 (SAS Institute, Cary, US).

## Results

### Baseline characteristics of study population

Overall, 30,171 patients from five different European countries (Germany, France, UK, Spain, and Italy) were included into this study. Baseline characteristics of the study population are given in Table 1. Patients had an average age of 65.9 (SD: 10.2) years, while the most frequent age group was 61–70 years (38.3%), followed by 71–80 years (27.3%), and 51–60 years (21.4%). The majority of patients were male (63.1%). 28.2% were treated in Germany, 21.2% in France, 18.9% in the UK, 17.5% in Spain, and 14.2% in Italy. 38.2% of patients were treated at academic cancer facilities, 24.7% at non-academic cancer facilities, 6.0% at general hospitals and 17.0% by office-based physicians. 48.4% were diagnosed with colorectal cancer, followed by pancreatic (21.9%) and stomach cancer (9.6%). 57% of patients had metastatic disease. Most patients were either symptomatic but fully ambulatory (61.2%) or asymptomatic (23.4%). First-line therapy (48.0%) and adjuvant therapy (23.8%) were the most frequent treatment settings within the study population.

**Table 1:** Baseline characteristics of study patients

Variable	Total
N	30,171
Age (mean, SD)	65.9 (10.2)
Age group (N, %)	
≤ 50	2069 (6.9)
51–60	6453 (21.4)
61–70	11,568 (38.3)
71–80	8233 (27.3)
> 80	1848 (6.1)
Sex	
Women	11,142 (36.9)
Men	19,029 (63.1)
Country	
France	6383 (21.2)
Germany	8515 (28.2)
Italy	4283 (14.2)
Spain	5278 (17.5)
UK	5712 (18.9)
Treating facility	
Academic cancer facility	11,510 (38.2)
Non-academic cancer facility	7452 (24.7)
General hospital	1813 (6.0)
Office based practitioner	5113 (17.0)
Unknown	4283 (14.2)
Key cancer	
Colorectal	14,613 (48.4)
Pancreas	6615 (21.9)
Stomach	2899 (9.6)
Liver	2705 (9.0)
Oesophagus	2178 (7.2)
Gastrointestinal stromal tumor (GIST)	1161 (3.9)
Current stage grade	
Localized	4728 (15.7)
Locally advanced	6431 (21.3)
Advanced	1811 (6.0)
Metastatic	17,201 (57.0)
Site of distant metastasis (most frequent)	
Liver	12,179 (40.4)
Peritoneum	5096 (16.9)
Lung	4857 (16.1)
ECOG performance status	
Asymptomatic	7055 (23.4)
Symptomatic fully ambulatory	18,450 (61.2)
Symptomatic in bed less than 50%	4297 (11.2)
Symptomatic in bed greater than 50%	331 (1.1)
Bedridden	38 (0.1)
Current line of therapy	
1st line advanced/metastatic	14,494 (48.0)
2nd line advanced/metastatic	3188 (10.6)
3d line advanced/metastatic	808 (2.7)
4th line advanced/metastatic	311 (1.0)
Adjuvant	7186 (23.8)
Neo-adjuvant	3527 (11.7)
Early stage/primary therapy	657 (2.2)

### Prevalence of treatment delay

**Fig. 1:**
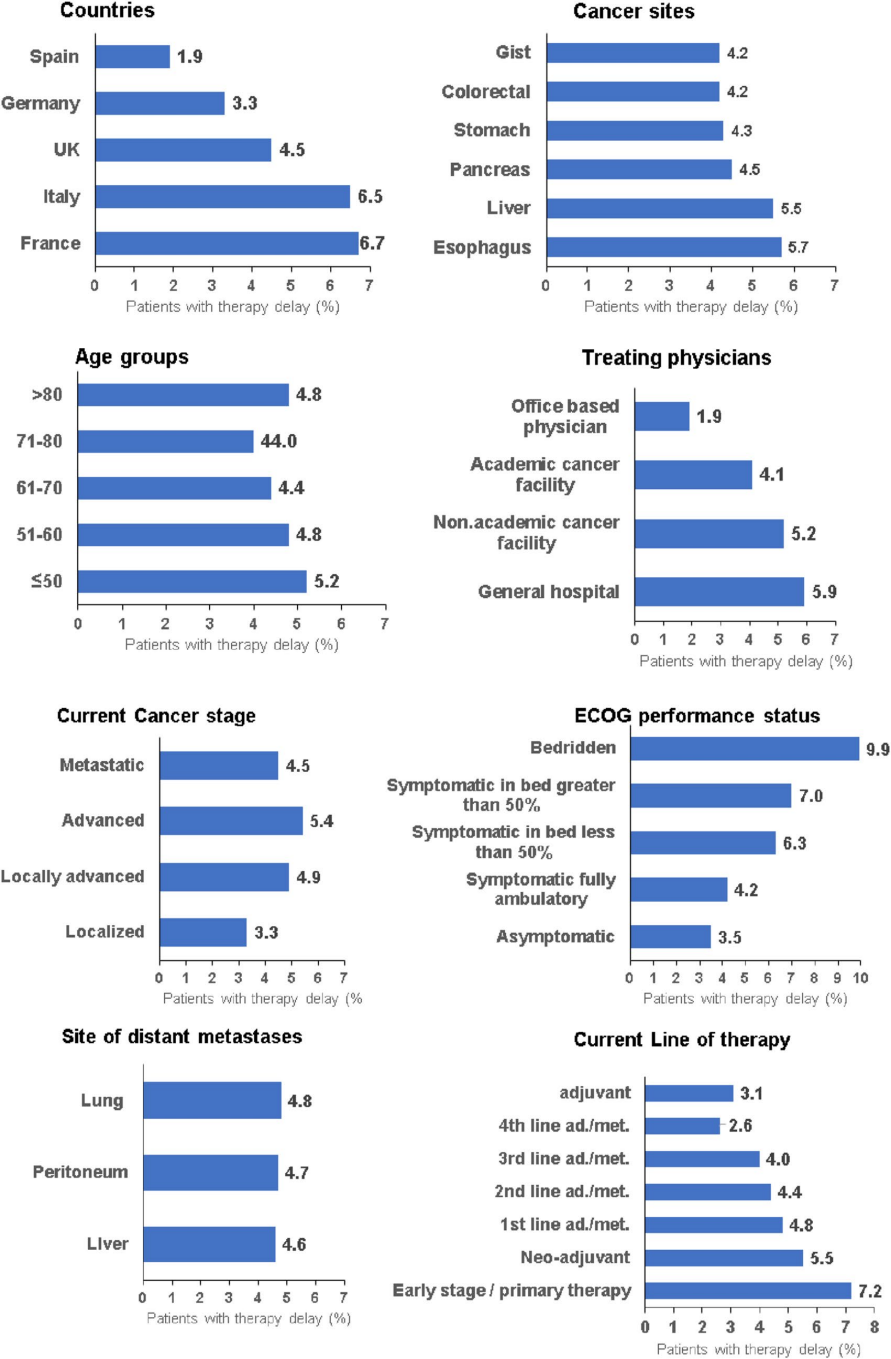
Prevalence of treatment delay by country, age group, cancer site, treating physicians, current cancer stage, ECOG performance status, site of metastasis, and current line of therapy

In a total of 1342 (4.5%) study patients, any treatment delay was documented. When a delay occurred, the treatment was most often postponed by less than 3 months (3.2%), followed by 3–6 months (0.7%). Rarely did the delay exceed 6 months (~ 0.5%). Figure 1 shows the prevalence of treatment delay as a function of the country, age group, cancer site, treating physicians, current cancer stage, sites of metastasis, ECOG performance status and current line of therapy, revealing distinct differences between the subgroups. For example, 6.7% of patients in France and 6.5% in Italy, but only 4.5% in the UK, 3.3% in Germany, and 1.9% in Spain, were documented to have experienced treatment delays (p < 0.001, Fig. 1). The prevalence of treatment delays differed slightly but significantly between cancer sites, ranging from 5.7% in esophagus and 5.5% in liver cancer patients to 4.2% in colorectal and GIST cancer patients (p = 0.008, Fig. 1). Importantly, 5.9% of patients treated at general hospitals but only 1.9% of those treated by office-based physicians experienced therapy delay (p < 0.001, Fig. 1). In terms of cancer stage, treatment delay was documented in 5.4% of patients with advanced cancer, followed by locally advanced cancer (5.4%), and 3.3% of patients with localized cancer (p < 0.001, Fig. 1). Moreover, the difference between lines of therapy was highly significant (p < 0.001), with 7.2% of patients in early-stage/primary therapy but 2.6% in advanced/metastatic cancer receiving 4th line therapy (Fig. 1). With respect to the ECOG performance status, the proportion of treatment delay increased from 3.5% in asymptomatic patients to 9.9% in bedridden patients (p < 0.001, Fig. 1). Finally, no significant differences between the age groups, sex, or between the three most frequent sites of metastasis were observed (Fig. 1).

### Results of multivariable regression model

Table 2 shows the results of multivariable logistic regression analyses for the association between predefined variables and treatment delay. We observed a strong association between treatment delay and the ECOG performance status, treating facility, country as well as the age group. With Germany serving as reference, Italy demonstrated the strongest association in treatment delays among the countries studied (OR 4.49; 95% CI 3.53–5.71). France was associated with a slightly increased prevalence of treatment delay (OR 1.31; 95% CI 1.09.1.57) and Spain with a strongly decreased prevalence of treatment delay (OR 0.34; 95% CI 0,26–0.43) when compared to Germany. With office-based physicians as the reference group, all hospital facilities were strongly associated with an increased prevalence of treatment delay with the highest OR in academic cancer facilities (OR 2.88; 95% CI 2.21–3.75). Compared with asymptomatic patients, the odds ratio for therapy delay increased from 1.84 (95% CI 1.56–2.17) for symptomatic, fully ambulatory patients to 3.69 (95% CI 1.09–12.50) for bedridden patients. Compared to the age group ≤ 50, the age groups 61–70 and 71–80 were associated with a lower therapy delay prevalence (Table 2).

**Table 2:** Association between treatment delay and demographic as well as clinical variables among GI cancer patients

Variable	Adjusted odds ratio (95% CI)	p value
Age group		
≤ 50	Reference	
51–60	0.88 (0.70–1.11)	0.289
61–70	**0.76 (0.61–0.94)**	**0.011**
71–80	**0.67 (0.53–0.84)**	**0.001**
> 80	0.75 (0.56–1.01)	0.056
Sex		
Women	Reference	
Men	0.98 (0.87–1.10)	0.733
Country		
France	**1.31 (1.09–1.57)**	**0.004**
Germany	Reference	
Italy	**4.49 (3.53–5.71)**	** < 0.001**
Spain	**0.34 (0.26–0.43)**	** < 0.001**
UK	0.87 (0.71–1.06)	0.174
Treating facility		
Academic cancer facility	**2.88 (2.21–3.75)**	** < 0.001**
Non-academic cancer facility	**2.79 (2.14–3.62)**	** < 0.001**
General hospital	**2.52 (1.82–3.49)**	** < 0.001**
Office based practitioner	Reference	
Key cancer		
Colorectal	Reference	
Pancreas	0.92 (0.79–1.07)	0.291
Stomach	0.89 (0.73–1.09)	0.262
Liver	0.99 (0.80–1.22)	0.902
Oesophagus	1.13 (0.91–1.40)	0.269
GIST	0.78 (0.56–1.09)	0.146
Current stage grade		
Localized	0.90 (0.66–1.25)	0.540
Locally advanced	1.22 (0.94–1.58)	0.145
Advanced	1.27 (0.98–1.66)	0.075
Metastatic	Reference	
Site of distant metastasis (most frequent)		
Liver	1.01 (0.85–1.19)	0.924
Peritoneum	1.09 (0.93–1.29)	0.291
Lung	1.06 (0.90–1.25)	0.509
ECOG performance status		
Asymptomatic	Reference	
Symptomatic fully ambulatory	**1.84 (1.56–2.17)**	** < 0.001**
Symptomatic in bed less than 50%	**2.70 (2.19–3.32)**	** < 0.001**
Symptomatic in bed greater than 50%	**2.03 (1.20–3.42)**	**0.009**
Bedridden	**3.69 (1.09–12.50)**	**0.036**
Current line of therapy		
1st line advanced/metastatic	Reference	
2nd line advanced/metastatic	0.95 (0.79–1.15)	0.601
3d line advanced/metastatic	0.72 (0.50–1.04)	0.076
4th line advanced/metastatic	0.47 (0.23–0.96)	0.038
Adjuvant	0.85 (0.65–1.11)	0.080
Neo-adjuvant	1.26 (0.97–1.62)	0.223
Early stage/primary therapy	1.42 (0.97–2.08)	0.072

Significant data are in bold (p < 0.05)

### Evolution of the prevalence of treatment delay over the course of the pandemic

**Fig. 2:**
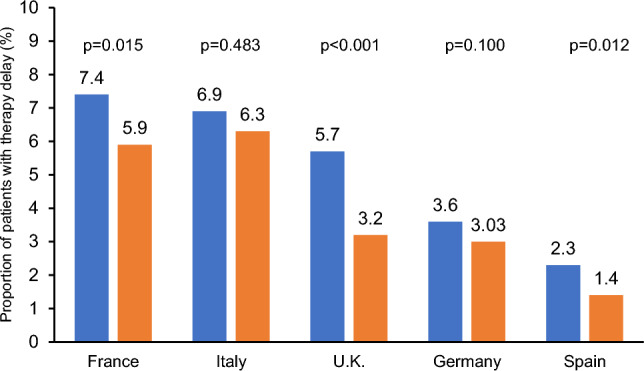
Prevalence of treatment delay by country and year (2021 vs. 2022)

Numerous factors with a potential influence on the prevalence of treatment delay have changed significantly over the course of the pandemic. These include non-pharmacological interventions, the popular perception of the situation, but also the possibility of vaccination and the development of various therapeutic options. We therefore compared the rate of treatment delays between the years 2021 and 2022- Fig. 2 shows the prevalence of treatment delay as a function of the country and year. Overall, the incidence of treatment delays decreased in all countries. Interestingly, this reduction was similar in all countries analyzed, albeit not statistically significant for Italy and Germany.

## Discussion

Previous studies have revealed a substantial impact of the COVID-19 pandemic on cancer care (Linjawi et al. 2023). Depending on the geography and study design, varying degrees of delays and cancellations of cancer treatment, screening and diagnosis have been observed, leaving the medium- and long-term impact on cancer care uncertain (Muka et al. 2023). By analyzing a total of 30,171 patients with gastrointestinal malignancies from five different European countries from IQVIA’s Oncology Dynamics (OD) database (Alymova et al. 2022), we show that for 1342 (4.5%) patients, a treatment delay occurred. The patients’ performance status, age, country of treatment, and treatment at general hospitals represented risk factors for treatment delays. Thus, our data highlight the tremendous effect of the COVID-19 pandemic on routine clinical cancer care even in high-income countries such as Germany, France, the UK, Spain, and Italy.

The COVID-19 pandemic has disrupted cancer care in several ways, likely leading to delays in diagnosis, treatment, and follow-up care (Teglia et al. 2022; Mazidimoradi et al. 2021). One of the most significant impacts of the pandemic has been the reduction in available medical resources, including healthcare professionals, medical supplies, and hospital beds. The pandemic has also led to delays in diagnostic tests, imaging studies, and surgeries, resulting in longer wait times for the patients (Teglia et al. 2022; Mazidimoradi et al. 2021; Teng et al. 2022; Dorri et al. 2021). Moreover, the pandemic has discouraged many people from visiting cancer treatment facilities for fear of contracting the virus (Muka et al. 2023). A recent umbrella review summarized and quantified the results of available systematic reviews on the impact of the COVID-19 pandemic on cancer treatment modification, delays, and cancellations (Muka et al. 2023), showing a decrease in screening rates across all cancer types. Globally, an overall reduction of 18.7% in the total number of cancer treatments administered from January to October 2020 compared to the previous periods was reported, with surgical treatment showing a greater reduction compared to medical treatment (− 33.9% versus − 12.6%), the largest reduction having been observed in skin cancer (Teglia et al. 2022). Treatment underuse and delays as well as interruptions in cancer care in general were more common in low- and middle-income countries (Majeed et al. 2022). In contrast to these previous analyses, our study is based on a cross-sectional, partially retrospective survey of anonymized patient cases from a representative panel of oncologists in 5 high-income European countries. We found treatment delays in 4.5% of cases, which is lower than most previously reported figures. Our analyses revealed the highest prevalence of treatment delays in Italy and France, while lower rates were reported in UK, Germany, and, unexpectedly, Spain, which was one of the most severely affected countries in Europe in terms of the pandemic. Regarding the different treatment facilities, treatment delays were more frequent in general hospitals and non-academic hospitals, while the lowest rates were found in office-based outpatient practices, which were probably able to adapt more efficiently to the SARS-CoV2 situation due to their smaller size and faster decision-making structures. The data suggest that telemedicine and remote consultations, which have become increasingly common in various aspects of cancer care such as treatment, screening, and rehabilitation, should be more widely implemented. Nevertheless, there is limited evidence on the positive and negative effects, as well as the cost-effectiveness, of telemedicine. Although some limited evidence suggests that telemedicine could reduce the costs of cancer care for patients and health care providers, there are concerns, particularly from patients, that telemedicine may not provide the same benefits as face-to-face consultations (Muka et al. 2023; Gundavda and Gundavda 2020).

A key finding of our study is that across Europe, patients with poor performance status were more likely to be affected by treatment delay, with bedridden patients (ECOG IV) at highest risk. This is of particular relevance as bedridden cancer patients are particularly vulnerable to the effects of the COVID-19 pandemic due to their compromised immune system and limited mobility. They are at a higher risk of severe complications and mortality. As a result, many of these patients are reluctant to seek medical care or undergo cancer treatment. In addition, healthcare facilities may not be equipped to adequately provide care for elderly and bedridden patients, which can lead to delays in treatment. Interestingly, multivariable analysis revealed that patients younger than 50 years of age were at higher risk of treatment delay, indicating that it was not a high calendar age of bedridden patients per se, but rather the extent of prior illnesses and individual mobility that determined inferior medical care during the COVID-19 pandemic. As the delay in cancer treatment could have a significant impact on cancer outcomes, these data raise the important question of inequalities in the delay or discontinuation of cancer treatment after SARS-CoV-2 infection. Notably, in line with our findings from Europe, race, ethnicity and area-level social determinants of health were associated with delayed or discontinued cancer treatment and longer delays in restarting drug-based therapies after SARS-CoV-2 infection in the US (Llanos et al. 2023). Llanos et al. concluded that *“multilevel interventions targeting microlevel and macrolevel determinants* (are needed) *to reduce the likelihood of delayed oncology care among vulnerable patient populations during public health emergencies”* (Llanos et al. 2023).

Similar to the patient-level factors, the line of treatment (adjuvant vs. first/ second line) was a strong determinant of a potential treatment delay. Patients receiving pharmacological therapy for the first time (i.e. neoadjuvant or adjuvant therapy or patients in the first palliative-intended line of therapy) had a significantly increased risk of treatment delay. This may be due to the fact that patients already known in practices/hospitals were treated preferentially and medical institutions tried to avoid new patients under the impression of the COVID-19 pandemic. On the other hand, it is precisely these groups that could most benefit from a timely intervention and who might therefore experience the most dramatic prognosis deterioration in the event of insufficient treatment. This observation can also play an important role in the efforts to be better prepared for future pandemic situations.

Of note, our study has important limitations. Some of these limitations are specific to cancer patients, whereas others reflect general limitations of the database as recently described (Loosen et al. 2022). Most importantly, it is possible that the database is not representative for the full spectrum of cancers. Regarding the database, it is important to note that only drug-treated patient cases are collected, and that the original questionnaire was not designed for the specific research purpose. Missing variables such as socioeconomic status represent further limitations. Moreover, the analyses don’t distinguish between delays due to the pandemic in general vs. delays due to the patients’ own infection. Finally, studies such as ours can only estimate associations, not causal relationships, and lack comparisons with other established databases. Nevertheless, the database has been used in numerous studies and has demonstrated its suitability for research purposes in several clinical analyses (Alymova et al. 2022).

In conclusion, our data highlight the problem of delayed treatment of cancer patients in the context of the COVID-19 pandemic. Identified risk factors for delayed treatment, such as poor general health or treatment in smaller hospitals, offer starting points for future concepts of “pandemic preparedness”.

## Data Availability

Data are available from the corresponding author upon meaningful request.
